# PI3K signaling through a biochemical systems lens

**DOI:** 10.1016/j.jbc.2023.105224

**Published:** 2023-09-09

**Authors:** Ralitsa R. Madsen, Alex Toker

**Affiliations:** 1 MRC-Protein Phosphorylation and Ubiquitylation Unit, School of Life Sciences, University of Dundee, Dundee, Scotland, United Kingdom; 2 Department of Pathology and Cancer Center, Beth Israel Deaconess Medical Center, Harvard Medical School, Boston, Massachusetts, USA

**Keywords:** PI3K, AKT, systems biology, growth factors, signaling

## Abstract

Following 3 decades of extensive research into PI3K signaling, it is now evidently clear that the underlying network does not equate to a simple ON/OFF switch. This is best illustrated by the multifaceted nature of the many diseases associated with aberrant PI3K signaling, including common cancers, metabolic disease, and rare developmental disorders. However, we are still far from a complete understanding of the fundamental control principles that govern the numerous phenotypic outputs that are elicited by activation of this well-characterized biochemical signaling network, downstream of an equally diverse set of extrinsic inputs. At its core, this is a question on the role of PI3K signaling in cellular information processing and decision making. Here, we review the determinants of accurate encoding and decoding of growth factor signals and discuss outstanding questions in the PI3K signal relay network. We emphasize the importance of quantitative biochemistry, in close integration with advances in single-cell time-resolved signaling measurements and mathematical modeling.

Faced with the remarkable complexity of cellular signaling networks, the human brain naturally turns to a reductionist approach that seeks to characterize smaller units, commonly known as signaling pathways, and their individual components. This approach has proven essential for understanding the pleiotropic physiological processes attributed to PI3K signaling as well as the consequences of disease-causing aberrations in individual pathway components. This reductionist biochemical and molecular approach to discovery resulted in the identification of multiple different catalytic and regulatory isoforms of PI3K, the biosynthetic routes of the key PI3K lipid products and second messengers PIP_3_ and PI(3,4)P_2_, and the discovery of effectors such as AKT and downstream substrates. As more and more information has accumulated on the molecular details underlying PI3K–AKT signaling, the field has begun to appreciate that the canonical PI3K pathway is embedded within a larger and highly context-dependent network of protein–protein, protein–lipid, protein–nucleic acid, and protein–metabolite interactions. As such, the intuitive view of PI3K signaling as a deterministic pathway is no longer sufficient to capture the complexity in the network (Fig. 1) and its role in human disease. This pathway-centric view is closely related to the excessive genocentric nature of modern biology, a limitation that was recognized by S. Brenner over 2 decades ago (1):

“…*we are drowning in a sea of data and starving for knowledge […] We need to turn data into knowledge and we need a framework to do so.*”

**Figure 1:**
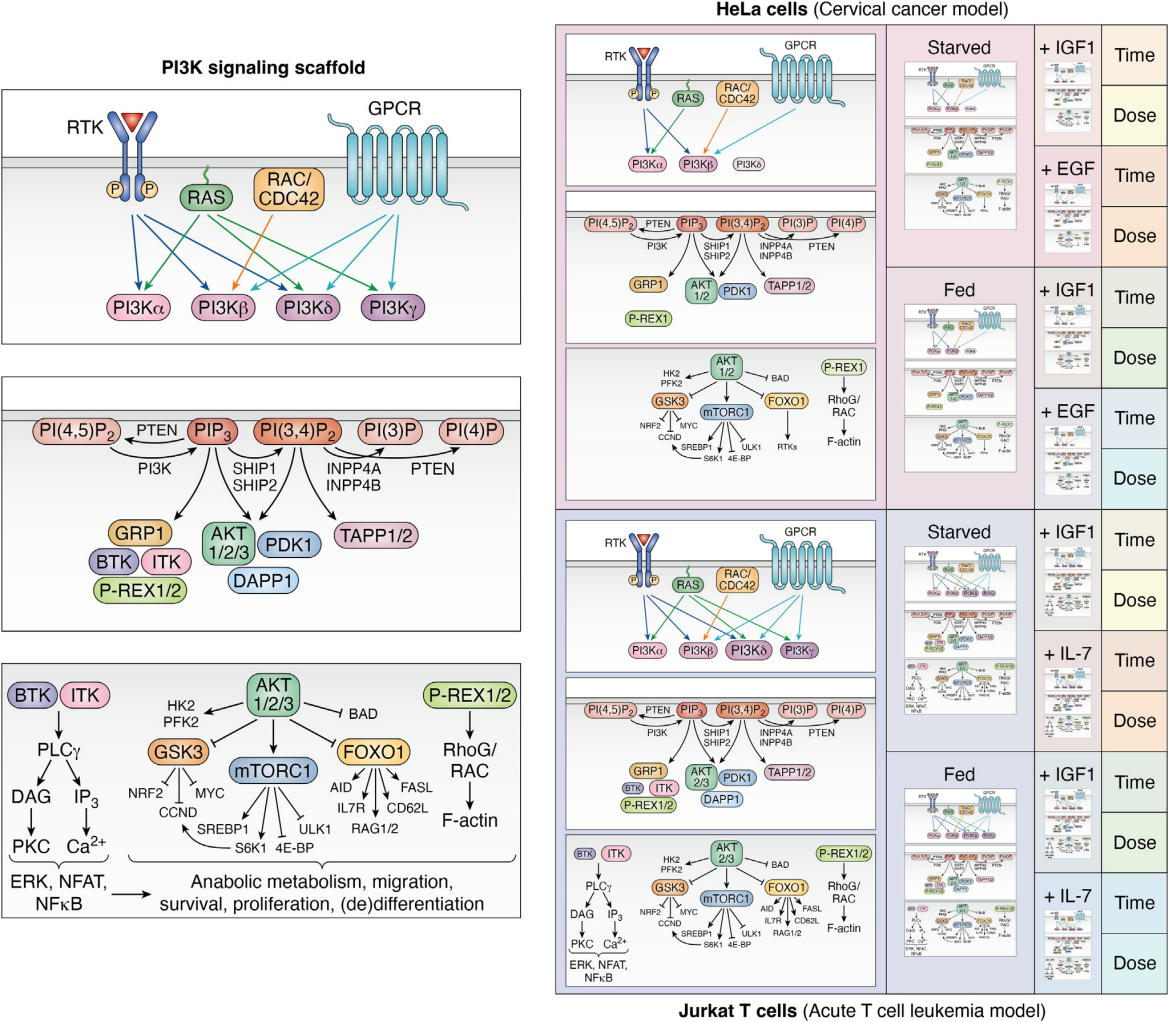
**Context-dependent PI3K signaling.** The same core PI3K signaling scaffold can be deployed differently in different cell type and lineages, exemplified here with commonly used adherent (HELA) and suspension (JURKAT T) cell line models and stimuli. The first set of subspaces show how the core PI3K signaling scaffold becomes modified because of cell line–specific expression of individual components. For example, HELA cells do not express BTK and ITK and have lower expression of PI3Kδ compared with JURKAT T cells. Within each cell line, multiple yet poorly understood instantiations of the modified PI3K signaling scaffold are possible depending on culture conditions and the application of specific stimuli. For example, the amplitude and/or cellular localization of a phosphorylated component may change as a function of the dose of and the time after a stimulus and may differ yet again for different stimuli. Cells use such changes in spatiotemporal PI3K signaling to achieve specificity in biochemical information transfer and downstream phenotypic control. For simplicity, this figure does not capture the full scale of the signaling network nor the extensive crosstalk with other classical pathways or the impact of non–cell-autonomous inputs. Created with BioRender and AffinityDesigner.

A solution to this problem was also originally posited by Brenner. It features biochemistry, cellular physiology, and the development of a quantitative framework in which to embed all prior mechanistic knowledge in order to compute outcomes of the complex interactions that result from the dynamics of the signaling system (2). Achieving this would require the ability to make parallel measurements of the behavior of all key signaling components while studying the execution of a cellular process over time (2). Although this would have been difficult to achieve 2 decades ago, the ensuing technological revolution within and outside biology has enabled the necessary tools for this challenge. Consequently, the biggest hurdle facing the PI3K signaling field at present is not the lack of tools and ideas but the many silos that exist and inadvertently preclude knowledge exchange.

In an attempt to address these challenges and de-silo the field, this review highlights poorly understood aspects of PI3K-dependent cellular information transmission, alongside examples of the interdisciplinary techniques and theoretical frameworks that are increasingly necessary for providing answers. These answers are, in our view, essential for future therapeutic advances in diseases of aberrant PI3K signaling. For reviews on classical PI3K and AKT signaling in physiology and disease, we point the reader to these recent articles: Refs. (3, 4, 5).

## From static cartoons to quantitative biochemical systems

A shared vocabulary is a prerequisite for interdisciplinary collaboration. An accurate definition of systems biology captures the essence of what S. Brenner offered as a solution to the big data problem. Systems biology is a scientific approach at the interface of physics, chemistry, and biology. The aim is an understanding of systems behavior, including concepts, such as homeostasis, feedback, and noise in biological systems (6). This, in turn, requires a firm basis in quantitative reasoning because of the need for precision in a system; the biology is equally essential because the ultimate goal is a precise understanding of biological function. Historically, systems biology is closely related to *physiology*, the original term used to define the study of system function in a biological context, whereas *anatomy* was reserved for descriptions of parts and their (static) connections (7). It is therefore unfortunate that systems biology in the present day is often used to refer to high-throughput data collection in static settings (*i.e.*, anatomy), in stark contrast to the legitimate quest for understanding the emergence of biological function from the dynamic interactions of individual components (*i.e.*, physiology) (8). Thus, simply having a big dataset of multimodal measurements is not sufficient for a study to classify as systems biology. Conversely, a detailed quantitative understanding of a single kinase-phosphatase cycle is systems biology. More specifically, two key conditions must be met for a systems biology qualifier: (1) the approach is quantitative and results in predictive models of system behavior and function over time; (2) the system itself is defined by specific boundaries, so that at first approximation, variables outside the boundary can be ignored (7).

That systems biology is firmly embedded in quantitative biochemical and molecular processes comes from the fact that cellular function emerges from chemical interactions among diverse molecules. Of these, proteins are the most important molecular machines and are linked functionally through allosteric or other mechanisms into biochemical relays. These relays carry out a range of computational tasks, including amplification, integration, and information storage (9). Consequently, our usage of static and pictorial representations of signaling networks belies the remarkable capabilities of these highly dynamic systems. These capabilities are ultimately determined by biochemical parameters.

The PI3K effector and serine/threonine kinase AKT serves as an ideal example to illustrate these concepts. The three AKT isoforms AKT1/AKT2/AKT3 are all capable of reading the cellular concentration of PIP_3_/PI(3,4)P_2_. Moreover, they facilitate the faithful interpretation of the spatiotemporal dynamics of PIP_3_/PI(3,4)P_2_, which can encode the identity of an upstream stimulus, such as growth factor–specific activation of receptor tyrosine kinases (RTKs). Depending on the quantitative properties of the upstream stimulus (*e.g.*, duration, strength), the downstream signaling and gene regulatory networks orchestrate an appropriate biological output. Importantly, the underlying biochemical circuits are not only responsible for enabling a cell to sense changes in its environment but also endow it with the capacity to anticipate such changes (10). Such cellular information processing is analogous to the information processing within the brain’s neural networks composed of hundreds of individual neurons that are subject to regulation in both time and space. While studies of individual isolated neurons are instrumental to understanding their fundamental properties, it is only through detailed and temporal studies of the entire neuronal network that principles of information transmission in the human brain can be inferred. Similarly, it is only through detailed temporal studies of the entire biochemical network that we can infer the rules that govern cellular information transmission. Such knowledge is necessary for the construction of quantitative models capable of generating testable predictions about specific biological mechanisms. This represents an iterative cycle between mathematical modeling and experimental hypothesis testing, the cornerstone of rigorously conducted studies in systems biology.

Within the PI3K signaling field, the best examples of systems biology approaches are found in foundational studies of the coupling between signaling pathways and cytoskeletal reorganization in the context of cell polarity and random and directed migration in eukaryotic cells. PIP_3_ is among the most upstream molecules to exhibit an asymmetric cellular distribution during chemotaxis and is also capable of inducing cell polarization and motility (10). Both PIP_3_ and its derivative PI(3,4)P_2_ form part of an exquisitely fine-tuned network of positive and negative feedback loops, including F-actin and members of the Ras and Rho GTPase families (11, 12). This so-called excitable system gives rise to self-organizing patterns featuring coordinated waves of PIP_3_ and F-actin-rich protrusions (11).

In the following sections, we focus on a much less well-understood aspect of PI3K signaling and one that would benefit from the same type of rigorous systems biology approaches used in the original studies of cell polarity and migration.

## An old question revisited: Achieving specificity in PI3K signaling

All PI3K isoforms are intracellular transducers of extracellular signals, including key growth factors, such as insulin, insulin-like growth factor 1 (IGF1) and epidermal growth factor (EGF). This extends beyond binary detection of a signal (present/not present) and includes the capture of dynamic concentration changes. This notion raises the question as to how cells achieve such discrete signaling specificity by using the same set of signaling molecules downstream of a wide array of cell surface receptors. While the field has yet to develop all the relevant context-dependent PI3K signaling models to address this question comprehensively and experimentally, the available evidence suggests that spatiotemporal (de)multiplexing is the basis for signal encoding through PI3K activation (5).

A key reason for our limited knowledge of quantitative input–output functions within the PI3K network is the technical challenge of precise and dynamic measurements of PI3K activation at the level of PIP_3_/PI(3,4)P_2_ biosynthesis (phospholipids found in miniscule amounts within cellular membranes), alongside the intracellular activities of key downstream effector enzymes such as AKT, ideally at single-cell resolution. Nevertheless, as discussed later, the currently available data across different signaling systems have provided important insights to build upon. While we focus most of the following discussion on the protein kinase AKT as one transducer of a growth factor–induced PI3K signal, additional PI3K and PIP_3_/PI(3,4)P_2_ effectors exist (13, 14, 15), and although understudied, likely utilize the same spatial and temporal constraints discussed later to enable high-fidelity information transfer.

### Temporal control

Bulk bioluminescence resonance energy transfer biosensor–based measurements of PIP_3_/PI(3,4)P_2_ production in live MCF7 breast cancer cells have revealed quantitatively distinct dynamics of PI3K activation, as a function of both stimulus identity and concentration (16). Remarkably, even 5 nM *versus* 1 nM IGF1 exhibit distinct PI3K activation kinetics. Their experimental capture requires high precision and temporal resolution (approximately every minute) as the difference is only apparent in the slope and magnitude of the signal in the first 10 min of growth factor stimulation, with subsequent activity levels converging at a similar quasi steady state (16). Thus, if the initial measurement is taken 10 min after stimulation, one would reach the erroneous conclusion that the two IGF1 concentrations are indistinguishable at the level of PI3K activation.

It is important to emphasize that there is currently no direct proof that such temporal differences in PI3K activity are used by cells to encode signal specificity, as observed for other signaling systems (5). A definitive proof requires a systems biology approach that combines quantitative biochemistry, phenotypic assays, and predictive mathematical modeling, with the latter guiding the choice of experimental perturbations for hypothesis testing in relevant biological models. Albeit limited in number, several independent studies have already made use of such approaches in the PI3K field, particularly in relation to understanding the encoding and decoding of insulin action at the level of AKT-dependent substrate regulation (17, 18). The data support nonlinear activation of AKT downstream of insulin, followed by demultiplexing of the signal. The latter is often achieved through differential engagement of AKT substrates, including dose–response alignment and kinetic regulation (17, 18). More recent studies have shown that pulsatile 0.1 nM insulin stimulation (akin to fasting insulin) of primary mouse hepatocytes rewires the kinetic response of AKT to subsequent treatment with 1 nM insulin (proxy for fed-insulin inputs), thereby selectively enhancing the response of a subset of substrates to physiological insulin stimulation (19). By contrast, repeated stimulation with 1 nM insulin (proxy for the hyperinsulinemic state) results in reduced AKT phosphorylation but increased extracellular signal–regulated kinase (ERK) phosphorylation (19). Such *in vitro* observations raise important questions about the regulatory mechanisms that underpin these differences in signal processing and whether they are relevant for understanding the selective activation of mitogenic responses in the context of metabolic insulin resistance *in vivo* (20).

**Figure 2:**
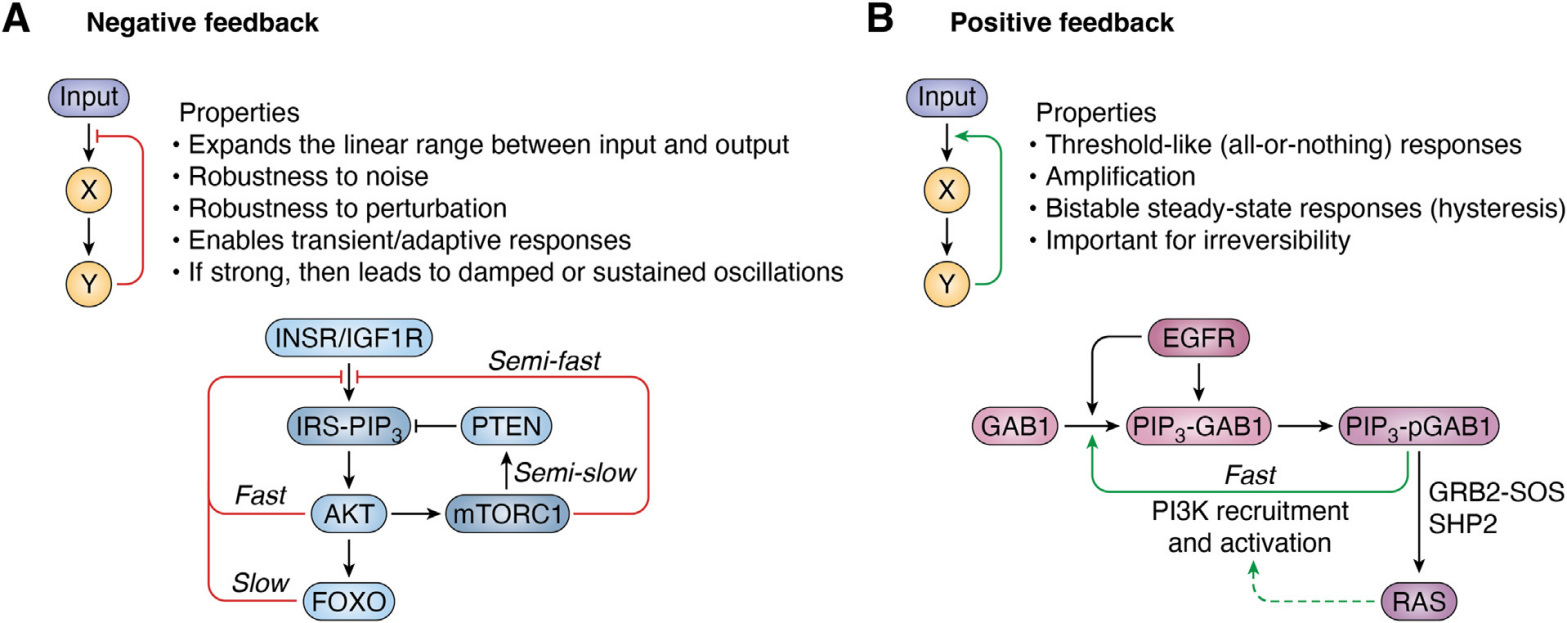
**Feedback control and dynamic signaling plasticity.** Feedback regulation is represented by two main motifs: negative (*A*) and positive (*B*) feedback loops. These endow signaling systems with different properties, examples of which are given in the respective *boxes*. For each regulatory motif, an example from the PI3K signaling network is given below. The example of negative feedback regulation reflects a synthesis of work presented in Refs. (93, 113, 114, 115, 116, 201, 202, 203, 204, 205, 206, 207, 208, 209, 210, 211). The example of positive feedback regulation was adapted from Ref. (165). Feedback motifs can operate at different time scales, as determined by the underlying molecular mechanism (*e.g.*, post-translational feedback mechanisms are faster than transcriptional feedback mechanisms). This, in turn, enables the precise tuning of the signaling response, which is used to specify a phenotypic outcome. While the examples in the figure indicate separate usage of positive and negative feedback motifs, the two can be combined to generate complex signaling functions. For example, mixtures of positive and negative feedback featuring integration of PI3K and RAS activity as part of an excitable network can be used to generate self-propagating cytoskeletal waves or cell polarization (11, 12, 212). Created with BioRender.

The existence of numerous feedback nodes within signaling networks, including PI3K, contributes to their temporal plasticity and capacity for dynamic encoding of specific stimuli (Fig. 2). The initial question how growth factor–specific responses arise from differences in intracellular signaling dynamics was raised in a classic study comparing EGF and nerve growth factor (NGF) responses in rat pheochromocytoma (PC12) cells (21). In this context, EGF triggers transient ERK activation and proliferation, whereas NGF triggers sustained ERK activity and differentiation (21). In the nucleus, the distinct duration of ERK signaling dynamics is decoded by early response genes such as the transcription factors c-Fos, Jun, Myc, and Egr1, which act as sensors for subtle changes in ERK activation and translate these into differences in cell state decisions (22, 23). In breast cancer cells, however, growth factors such as EGF and heregulin induce transient *versus* sustained ERK *cytoplasmic* activity, yet *nuclear* ERK activity is transient in either case (24). Integrated computational and experimental approaches have identified that the underlying mechanism resides in a robust transcriptional negative feedback loop, triggered primarily by a subtle yet significant growth factor–specific difference in cumulative nuclear ERK activity within 15 to 60 min of stimulation (24). Because of this negative feedback, heregulin—but not EGF-treated cells—become refractory to further ligand stimulation at the level of *c-Fos* expression (24). Overall, this example illustrates a temporal control mechanism used by cells to achieve spatial coordination of signaling activities. Ostensibly, this represents a reversal of the relationships covered in the following examples of spatial control of temporal signaling activity. There is no *a priori* reason to assume that such control mechanisms would apply only to RAS–ERK signaling, and our expectation is therefore that they await to be discovered in the PI3K signaling network. We further anticipate that such work will benefit from the use of spatially restricted biosensors and location-selective inhibition of signaling targets (25, 26).

### Spatial control

Differences in adaptor protein recruitment to activated RTKs contribute another layer of signal diversification by controlling the intracellular trafficking and thus the spatial regulation of activated receptors. Restricting the signaling machinery to particular cellular compartments naturally provides increased ligand sensitivity as well as specificity in the downstream responses. For example, the probability of ligand–receptor binding will be higher in the restricted confines of endosomal vesicles. Moreover, internalized receptor activation also facilitates rapid signal delivery to relevant subcellular locations and thereby triggers qualitatively and quantitatively distinct signaling events (27). Although we focus the following discussion on epidermal growth factor receptor (EGFR) and insulin-like growth factor 1 receptor (IGF1R)–insulin receptor (INSR) because of more complete mechanistic understanding of these systems, the salient features of the illustrated examples extend to other RTKs, including vascular endothelial growth factor receptors (28), angiopoietin receptors (29), and platelet-derived growth factor receptors (PDGFRs) (30, 31), to name a few.

#### EGFR

The EGFR represents the prototypical model receptor for spatial regulation of signaling specificity. EGFR can be activated by seven distinct ligands known to produce quantitatively, and to a lesser extent, qualitatively distinct signaling responses (32). EGFR is also only one of four members of a family of related receptors (EGFR, ERBB2/HER2, ERBB3/HER3, and ERBB4/HER4), yet only EGFR and ERBB4 are capable of forming signaling-competent homodimers upon ligand binding, whereas all members can form various heterodimeric combinations (33). Together with studies of RAS–ERK, our quantitative understanding of EGFR signaling is more advanced than for many other systems, in large part because of early efforts to address key questions in this field through a *bona fide* systems biology approach (34, 35, 36, 37, 38, 39, 40).

The sensing of variations in the strength and timing of growth factor signals is enabled through coordination of EGFR activation kinetics, endocytosis of receptor complexes and subsequent vesicular trafficking, as well as spatially restricted phosphatase-mediated signal termination (41). Collectively, these mechanisms ensure rapid amplification of EGFR phosphorylation at low growth factor concentrations and the ability to reset to baseline when growth factors decline. Concurrently, the system is also endowed with the capacity for transient biochemical memory of stimulus history (41, 42, 43). Upon activation, the EGFR undergoes rapid endocytosis and either recycles back to the plasma membrane or commits to endosomal signaling on its way to lysosomes where the receptor is degraded as part of signal termination. The choice between the two internalization fates is mediated by a ubiquitylation threshold, which is itself causally determined by the extent of EGFR tyrosine phosphorylation *via* recruitment of the E3 ubiquitin ligase Cbl (44). Detailed quantitative biochemistry and mathematical modeling studies have revealed that this system exhibits complex behavior as a function of ligand and receptor concentrations (45). The system is optimized to respond in the physiological range of EGFR levels (spanning one order of magnitude), with increasing concentrations of EGF promoting receptor degradation to limit overstimulation. This relationship no longer holds at supraphysiological levels of EGFR expression, because of limiting levels of Cbl (45). Under these conditions, the mathematical model predicted and resulting data have confirmed a progressive uncoupling of EGFR phosphorylation and ubiquitylation, an intrinsic weakness that appears to be exploited by cancer cells harboring EGFR overexpression (45).

The distinct EGFR internalization mechanisms elicit different signaling outputs and are subject to context-dependent regulation. It has been suggested that activation of PI3K–AKT by EGFR and other RTKs may be necessary for biochemical memory and sensing of time-varying signals (43), mediated by AKT-induced receptor recycling (46) as well as PI3K-dependent activation of NADPH oxidase (47, 48). The resulting local generation of reactive oxygen species causes reversible inactivation of several phosphatases at the plasma membrane, in what constitutes a double-negative feedback motif that maintains growth factor responsiveness (42). Conversely, cell contact–induced activation of Ephrin receptor downregulates AKT phosphorylation and EGFR recycling, whilst allowing continued RAS–ERK signaling from early endosomes (49). Thus, by inhibiting EGFR recycling downstream of Ephrin receptor signaling, increased cell density impairs a spatially restricted positive feedback that generates elevated PI3K–AKT activity required for continued exploratory behavior upon persistent EGF stimulation (49). It is important to note, however, that the proposed spatial restriction of AKT activity requires further confirmation as the existing evidence is based on the use of the isolated PH domain of AKT1, which is representative neither of the localization nor of the activity of the full-length protein kinase (50, 51).

#### IGF1R–INSR

Activation of IGF1R typically causes sustained tonic activation of PI3K–AKT signaling. Studies in rat L6 myoblasts have revealed an underlying mechanism that depends on insulin receptor substrate-1 (IRS1)–mediated suppression of the AP2 clathrin adaptor protein and an associated delay in IGF1R internalization (independent of the conventional role of IRS1 as a phosphotyrosine adaptor protein) (52). Accordingly, depletion of IRS1 results in accelerated IGF1R endocytosis, a switch from sustained to transient AKT phosphorylation, and attenuated suppression of forkhead box O (FOXO)–mediated transcription (52).

Additional evidence indicates that the difference in the relative balance between PI3K–AKT *versus* RAS–ERK activation underpins the functional classification of the IGF1R as “mitogenic” compared with the highly homologous INSR, which is classified as “metabolic.” This relative balance appears to be determined by the extent and route of receptor internalization (53). Notably, a substitution of a phenylalanine for leucine at position 973 in the INSR (numbering relative to the +exon 11 B isoform), making it equivalent to the corresponding residue in the IGF1R, results in IGF1R-like signaling and phenotypic responses downstream of INSR activation in brown preadipocytes and differentiated adipocytes (54). This switch is accompanied by impaired INSR internalization, once again mimicking the prolonged cell surface retention known to characterize IGF1R. Although it is suggested that the mitogenic response downstream of the phenylalanine for leucine at position 973 substitution is primarily a result of decreased binding and/or activation of IRS1–PI3K–AKT in favor of SHC–RAS–ERK, it is also tempting to speculate that the impaired internalization of the mutant INSR may alter the kinetics of PI3K–AKT activation and thereby contribute to a more IGF1R-like phenotypic response (52, 54). Further quantitative studies are needed to test this hypothesis, which may also be relevant for the aforementioned rewiring of signaling kinetics upon pulsatile stimulation with 1 nM insulin (19) as well as the more mitogenic action of isoform A (INSR-A) of the INSR and of hybrid IGF1R–INSR complexes (55).

The phenotypic specificity of different ligands acting through the same receptor may also rely on a differential regulation of receptor internalization. For example, compared with insulin, IGF2 has lower affinity for INSR-A and is less potent at activating downstream effectors, yet causes a more sustained mitogenic response, which has been attributed to reduced receptor internalization (56). A combination of cell biology, quantitative signaling studies, and computational modeling has also shown that mouse embryonic fibroblasts (MEFs) with loss of imprinting (LOI) of IGF2, and thus higher levels of as well as increased sensitivity to the ligand, exhibit a rebalancing of PI3K–AKT *versus* RAS–ERK signaling downstream of IGF1R because of differences in receptor trafficking (57). The sustained tonic phosphorylation of AKT primarily occurs at the cell surface. By contrast, the transient induction of RAS–ERK activation relies on efficient receptor endocytosis and exhibits an increased fold change in LOI MEFs, concurrently with a lower amplitude of AKT phosphorylation. Altogether, LOI MEFs exhibited enhanced ERK-dependent proliferation and higher dependence on IGF1R-mediated activation of AKT for cell survival, which could potentially be exploited for selective targeting of the mutant cells (57).

#### Beyond receptor trafficking

Spatial control of signaling output is mediated by additional mechanisms beyond differential receptor trafficking. For example, PIP_3_, the most proximal output of PI3K activation, is dephosphorylated to either PI(4,5)P_2_ or yet another second messenger, PI(3,4)P_2_ (58, 59). The latter can also be produced independently of class I PI3K activity and has important roles in regulation of the cytoskeleton and endocytosis (60). Endosomal PI(3,4)P_2_ contributes to sustained AKT signaling following growth factor stimulation (51, 61), with evidence demonstrating selective control of the AKT2 isoform in this context (51, 62).

Spatially insulated signaling complexes may also form upon liquid–liquid phase separation–like processes. RTKs such as EGFR are known to form highly localized condensates upon activation, because of recruitment of adaptor proteins to the phosphorylated receptors. This brings the relevant enzymes and substrates in close proximity while simultaneously minimizing access to unintended targets (63). The dynamic formation of INSR clusters across the cell has also been shown to characterize insulin-sensitive cells but not insulin-resistant cells (64). *De novo* membraneless RTK-based pathogenic biomolecular condensates have recently emerged as alternative sites of oncogenic activity in cancer cells (65), further emphasizing the importance of considering liquid–liquid phase separation in spatiotemporal signaling regulation.

Finally, intracellular phosphorylation gradients are also likely to emanate from the differential localization of specific phosphatases within the PI3K network, including phosphatase and tensin homolog on chromosome 10 (PTEN), inositol polyphosphate 4-phosphatase type II (INPP4), and PH domain leucine-rich repeat protein phosphatase (PHLPP) (42). This is an area that has received relatively scant attention, yet is likely to be critical for understanding subcellular regulation of PI3K signaling.

### Biochemical determinants

**Figure 3:**
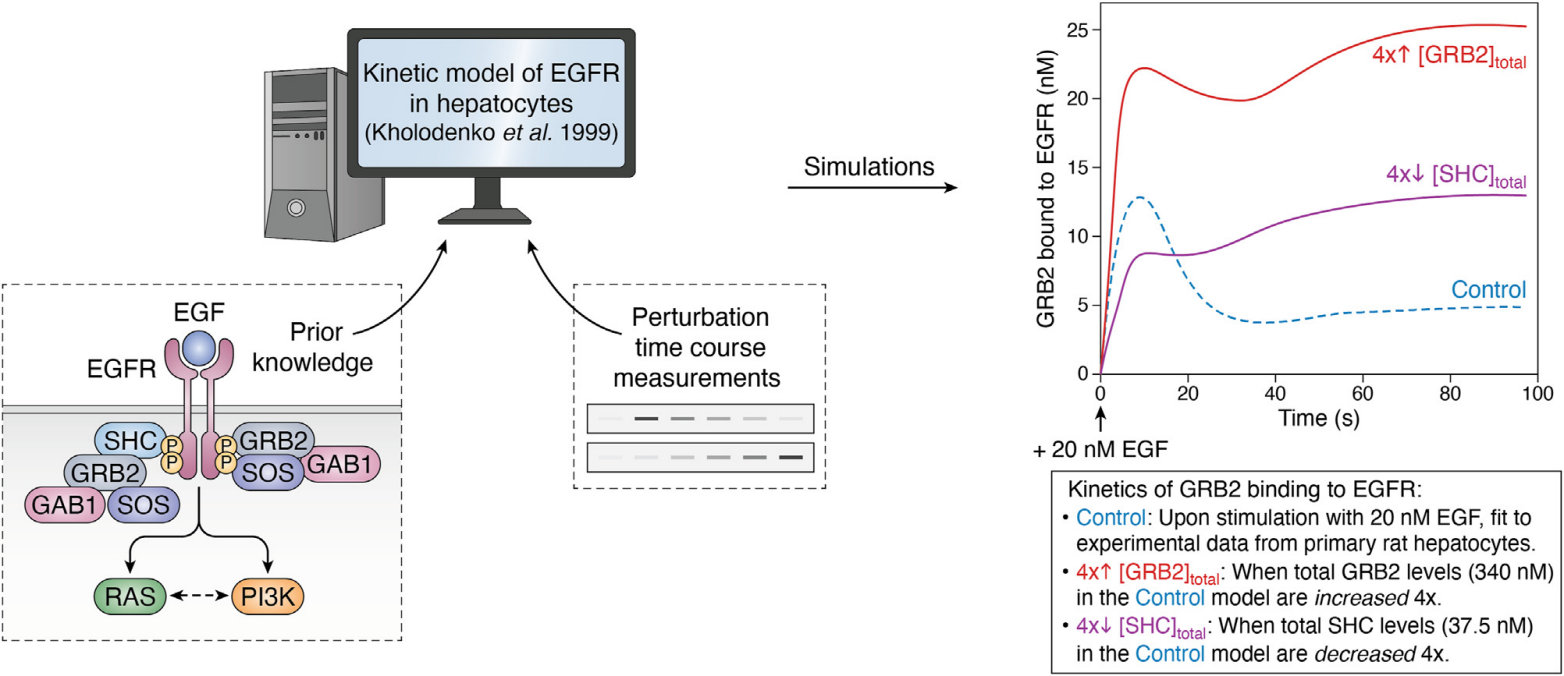
**Biochemical determinants of dynamic signal encoding.** Available knowledge of signaling topology and biochemical parameters (rate constants, affinity constants, and concentrations) can be used to construct predictive mathematical models of signaling phenomena, provided that such models are calibrated with the relevant experimental data. Shown here is how one of the first models of short-term epidermal growth factor (EGF) signaling in primary hepatocytes could be used to simulate the nonlinear kinetics of GRB2 recruitment to the activated EGF receptor (EGFR) as a function of concentration changes in GRB2 itself or SHC. GRB2 and SHC represent key adaptor proteins for activation of RAS–ERK and PI3K signaling downstream of EGF. Created with BioRender and adapted from Ref. (34). ERK, extracellular signal–regulated kinase.

In addition to the overall network structure, the subcellular localization, and temporal activity of its individual components, the development of predictive mathematical models of PI3K signaling will require estimates of the key biochemical parameters that determine information flow within the network. These parameters include concentrations of individual components (*e.g.*, ligands, receptors, adaptor proteins, kinases, and phosphatases), initial activity status, reaction rate constants, and diffusion coefficients. Figure 3 illustrates how a kinetic model of EGFR signaling has been used to predict the dependence of the temporal response to EGF on the concentration of key adaptor proteins (34). This may be subject to cell type–specific regulation and thus contribute to heterogeneous context-dependent signaling responses (66). A kinetic model of PI3K signaling has also shown that the concentration of the mechanistic target of rapamycin complex 2 (mTORC2)–specific component stress-activated protein kinase–interacting protein-1 underpins a biphasic relationship between mTORC2–AKT and mTORC1 activities, in contrast to the common assumption of a positive linear link (67).

These examples demonstrate how nonlinear signaling dynamics can arise because of seemingly simple biochemical changes, in the absence of complex feedback loops. Consequently, it is easy to conceive how such changes may result in altered information transmission in a cell with a disease-causing mutation in a key signaling kinase such as PI3Kα, one of the most frequently mutated components in human cancers. As precise biochemical descriptions of activating mutations in the canonical PI3K pathway are uncovered (68, 69, 70, 71, 72), it will be important to capture these in mathematical models of growth factor–specific signaling. Computational simulations can then be used to predict and subsequently validate how distinct mutations recode cellular information processing, thereby facilitating context-aware classification of such variants, with obvious therapeutic implications (5, 73, 74). In principle, this could be extended to model the effect of disease-causing mutations throughout the signaling network and to predict the complex responses to different pharmacological PI3K pathway inhibitors.

Differences in biochemical determinants also underpin the relative usage of shared signaling components downstream of distinct receptors. Accordingly, when six RTKs are stably overexpressed in the same cellular background, they activate many of the same proteins but to different degrees because of quantitative differences in both the number of docking sites and their binding affinities (75). Although both IGF1R and PDGFRβ feature docking sites for the p85 regulatory subunit of class IA PI3K, the single docking site on IGF1R is of low affinity (*K*_*D*_ = 590 nM), whereas PDGFRβ has five docking sites including a high-affinity site (*K*_*D*_ = 10 nM) (75). These biochemical quantities can be incorporated into statistical models capable of predicting the relative phosphorylation levels of upstream signaling proteins. This has revealed that for six different RTKs (IGF1R, PDGFRβ, EGFR, FGFR1, MET, and NTRK2), the variables with the largest contribution to the predictive power of the model is the number and affinity of docking sites for the adaptor proteins SHC1 and p85α (75). If binary information is used alone (proteins either interact or do not interact with an RTK), the prediction fails since all six receptors are capable of recruiting similar adaptors, making it impossible to differentiate receptor-specific signaling based on qualitative information alone (75).

While useful for understanding signaling differences across RTKs, this example does not explain how the same RTK can recruit different sets of downstream effectors as a function of ligand dose. Studies have shown that low-*versus*-high concentration of EGFR ligands elicit distinct and sometimes opposite phenotypes (*e.g.*, higher *versus* lower cell proliferation) (76, 77, 78). There is evidence to suggest that distinct EGFR signaling modes downstream of different concentrations of the same ligand are controlled by the balance of EGFR dimer *versus* oligomer formation (79). Furthermore, low- *versus* high-affinity EGFR ligands stabilize different dimeric receptor conformations, which in turn determine the observed ligand-specific spatiotemporal kinetics of PI3K as well as RAS–ERK signaling (80). Notably, *weaker* EGFR dimerization, as elicited by low-affinity ligands such as epiregulin and epigen, results in more sustained downstream signaling compared with EGF and other high-affinity ligands, and causes distinct phenotypes (80). The underlying molecular mechanism may constitute a form of kinetic proofreading whereby short-lived EGFR dimers do not reach the end of a progressive multisite phosphorylation wave and thus fail to elicit all possible signals including recruitment of phosphatases and/or endocytic adaptors (80). Attesting to the importance of this structure-dependent phenomenon, glioblastoma mutations that alter EGFR dimer formation attenuate ligand bias and thereby corrupt downstream signaling specificity (81).

Collectively, these examples emphasize that mapping key biochemical determinants is essential as part of integrated workflows aimed at generating a quantitative systems-level understanding of the PI3K signaling network.

### Population *versus* single-cell responses

**Figure 4:**
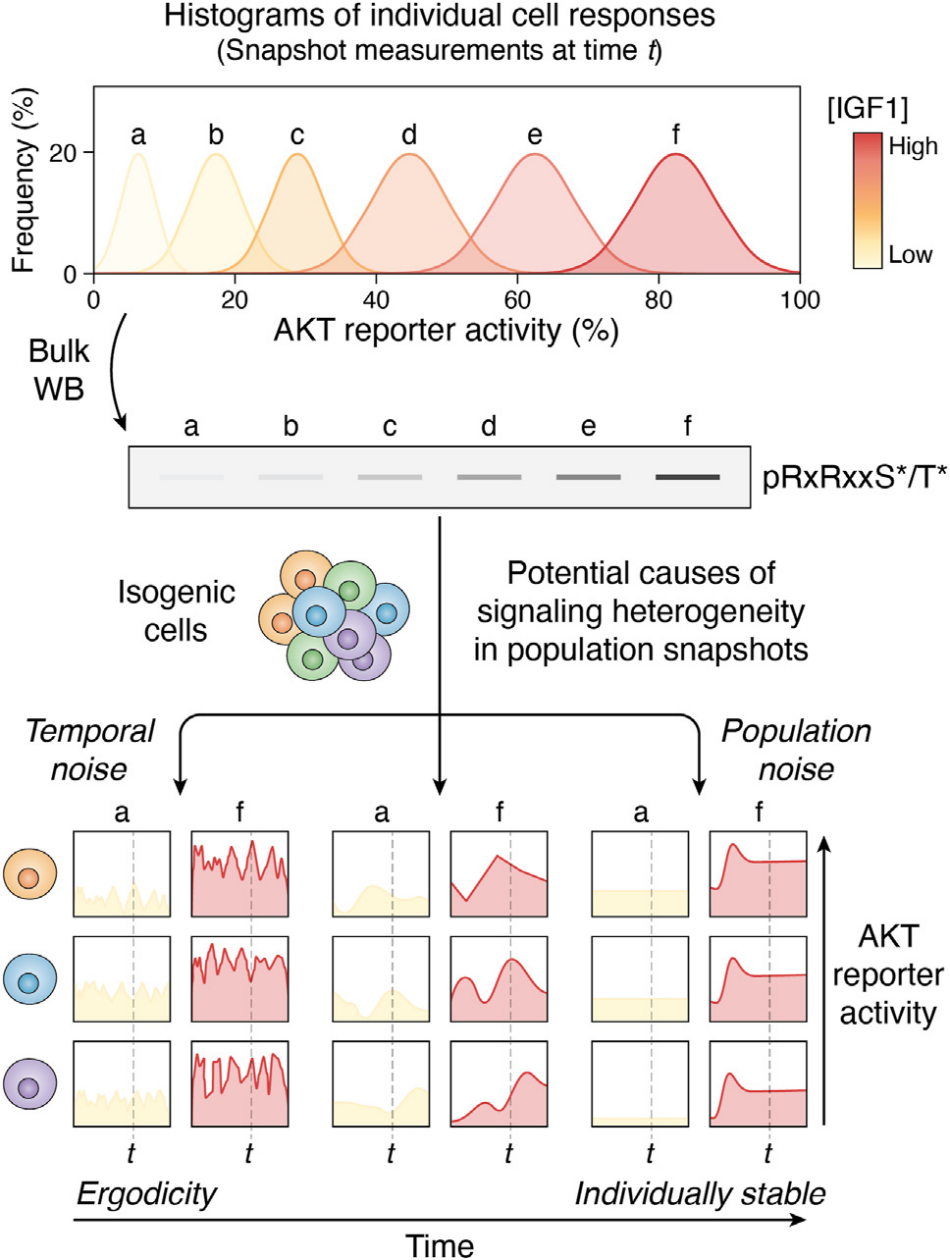
**Single-cell heterogeneity and limitations of snapshot signaling measurements.** Across an otherwise isogenic cell population treated with an increasing concentration of IGF1, a snapshot measurement of AKT activity will reveal a wide response distribution at the single-cell level (86). This heterogeneity is obscured by bulk immunoblotting for AKT substrate phosphorylation. Population snapshot measurement cannot reveal whether the observed response heterogeneity is caused by high intracellular response variability, high intercellular variability with otherwise stable individual responses, or a mixture of both. To resolve this, one needs dynamic signaling measurements at high temporal resolution, particularly in the case of an ergodic system (one where the time average of a rapidly fluctuating signaling response in a single cell equals the population average in a snapshot measurement). Live-cell measurements of AKT reporter activity in single cells suggest that individual cell responses are stable, with population heterogeneity arising from a mixture of stable high and low responders (86). Such low intracellular variability in PI3K–AKT signaling may enable individual cells to encode extracellular signals reliably. Created with BioRender based on Refs. (86, 97). IGF1, insulin-like growth factor 1.

To date, most studies on PI3K signaling have been conducted in bulk cell populations, whereas relatively few have evaluated single-cell responses. Advances in single-cell technologies now permit such experimental considerations (82, 83, 84). Bulk population measurements obscure the substantial single-cell heterogeneity that characterizes PI3K–AKT signaling responses (85, 86, 87) and may therefore confound efforts to build predictive models of the underlying quantitative input–output relationships (Fig. 4). Unlike the frequency-modulated (*i.e.*, digital “all-or-nothing”) pulses of ERK activity used by single cells to encode increasing growth factor concentrations (88), single-cell studies in distinct cell types expressing genetically encoded biosensors for PI3K or AKT activity indicate a more analog nature of signal transmission (86, 87, 89, 90, 91, 92, 93). Thus, at least in synchronized cells, increasing doses of a growth factor result in gradual activation of PI3K signaling components, according to highly reproducible patterns within the same cell (intracellular signaling). Yet, responses across an otherwise isogenic population of cells (intercellular signaling) are highly heterogeneous (Fig. 4) (86, 87, 89, 90, 91, 92, 93).

The mechanistic basis for differences in quantitative signaling determinants in a cell population is starting to be investigated. A multiplexed, spatially resolved, single-cell quantification of signaling in the context of acute stimulation with EGF revealed that signaling responses are conditioned on cellular state (*i.e.*, intrinsic and extrinsic physicochemical properties) (94). The latter had a stronger effect on growth factor responses than changes in growth factor concentration. This suggests that a signaling network functions as a multimodal percept capable of capturing partially nonredundant information about cell state and, in turn, of executing cell state–dependent phenotypic decisions (94). The technical term for this phenomenon is *adaptive information processing* and may have coevolved with complex signaling networks to enable context-aware decision making in a multicellular setting (94). It is perhaps no coincidence that this type of adaptive information processing is also considered the operational feature of real as well as artificial neural networks.

The previous example is consistent with the notion that the same signaling network configuration can display multiple stable solutions or what is commonly referred to as “attractors” in physics (95). This is an important concept that relates directly to phenotypic heterogeneity and is particularly relevant for understanding cancer cell plasticity and resistance to targeted therapies (95, 96, 97, 98, 99). Attractor landscapes have primarily been considered in relation to epigenetic and transcriptional regulation because of the relative ease with which one can obtain high-throughput single-cell sequencing datasets. Theoretical and technological advances are now enabling equivalent quantitative landscape maps to be generated for signaling networks and integrated with the corresponding epigenetic and transcriptomic landscapes. Yet, unlike the classic view of a deterministic Waddington landscape where cells visualized as balls roll down static valleys, there is now substantial evidence that the valleys (*i.e.*, molecular attractors) themselves change as a function of environmental inputs, non–cell-autonomous processes, and regulated noise (100, 101, 102). The implications of such nonstatic quantitative cell state mapping are profound and have been the subject of several excellent reviews (96, 100, 101, 103). In the following, we will focus on two key points that are directly relevant for studies of PI3K signaling in health and disease.

### The importance of noise in cellular decision making

**Figure 5:**
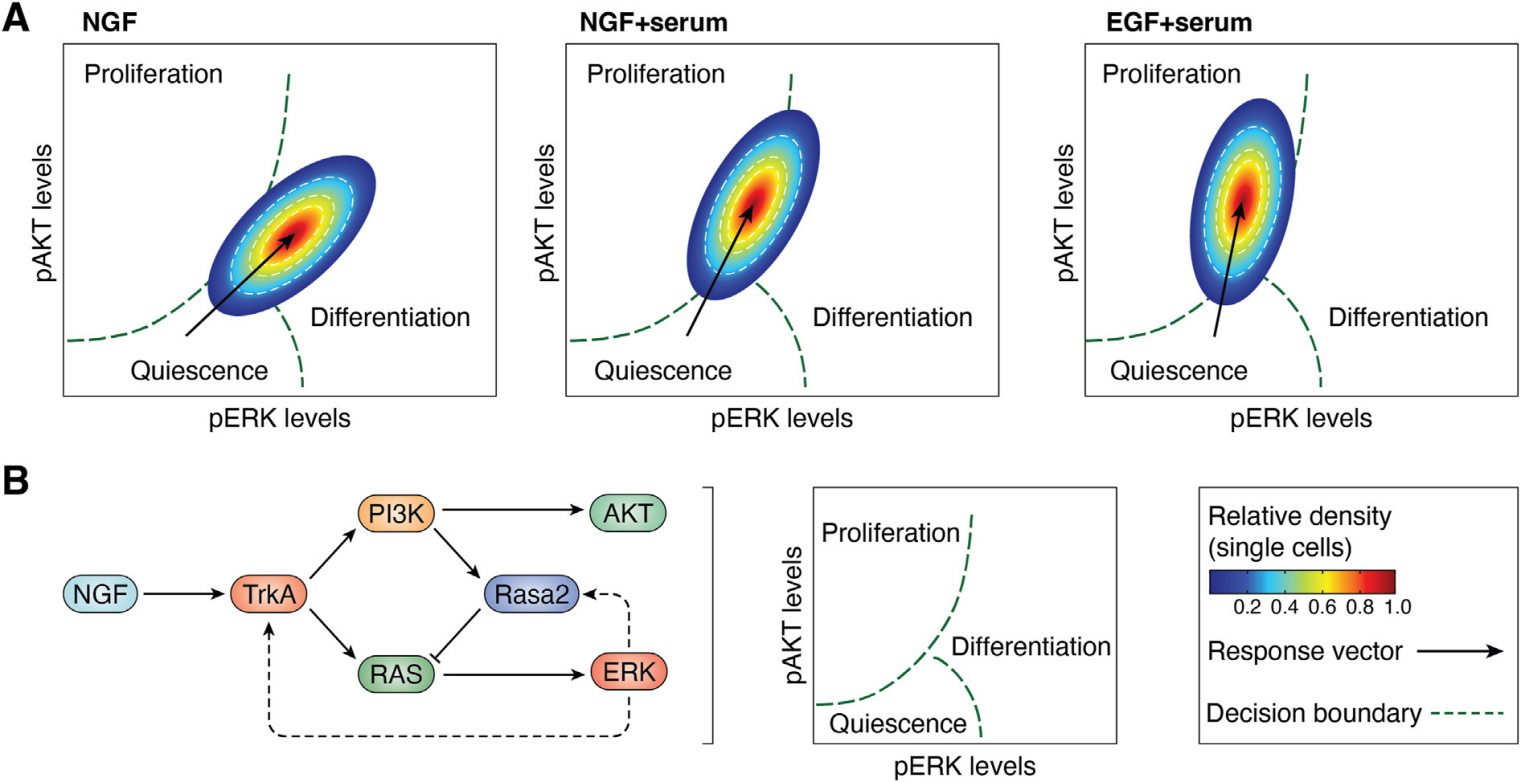
**Probabilistic signaling response maps.***A*, when rat pheochromocytoma (PC12) cells are stimulated with neural growth factor (NGF) *versus* epidermal growth factor (EGF), they exhibit differences in their propensity for differentiation *versus* proliferation. Quantitative single-cell analyses have revealed the existence of a sharp nonlinear decision boundary governed by a two-dimensional pAKT–pERK plane. PC12 cells with a higher probability of proliferation have higher pAKT and lower pERK, and vice versa for cells that are more likely to differentiate. For each perturbation, a response vector is drawn to indicate the direction of the population shift. The position of the cell population along the decision boundary is determined by a feedback mechanism that involves PI3K signaling, RAS–ERK signal modulation, and the Ras GTPase-activating protein (GAP) Rasa2 (*B*). Combined with stochastic variation in protein expression levels of individual signaling components, this constellation ensures that the same stimulus can give rise to coexisting cell populations with distinct phenotypes, according to specific probabilities. Both (*A* and *B*) are adapted from Ref. (106) and represent approximations of the original plots. ERK, extracellular signal–regulated kinase.

First, noise within the PI3K signaling network, and in cell biology more generally, is both a *regulated* property and a *regulatory* property. It shapes and is shaped by cellular responses (100, 101, 102). It is a mechanism like any other, selected and optimized by evolution because of its importance for survival in fluctuating environments, so-called “bet hedging” (104). Noise is inherent to any regulatory network operating within confined compartments and with limited numbers of constituent molecules. Theoretical considerations have even suggested that biological noise enables cellular populations to execute complex functions despite the use of relatively simple signaling machineries (105). The existence of such noise moreover implies that predictions of input–output relationships in relation to PI3K signaling can only be of a probabilistic nature. A response map of this kind was already generated by the Meyer group 11 years ago, based on single-cell measurements of phospho-AKT, phospho-ERK, and cell proliferation in PC12 cells stimulated with NGF *versus* EGF (106) (Fig. 5). This represents a shift away from a deterministic, and overwhelmingly static, view of PI3K signaling and cellular regulation, toward the development of probabilistic landscape maps that integrate multimodal measurements of single-cell states (96). Such maps will represent probabilities of cell-specific responses following systematic perturbations. This could, for example, be used to train computational models that predict the pharmacological interventions or genetic interventions that increase the probability of a particular phenotypic change (107). This probabilistic view also has the potential to reconcile apparent discrepancies in the literature, where mutually exclusive responses have been associated with PI3K signaling (5). It is entirely possible that these do co-occur within a cellular population but according to different context-dependent probabilities (108). Thus, because of the power of statistics, the same noise that makes individual cell behavior hard to predict may be responsible for accurate cellular decision making and division of labor across a cell population (100, 106), as required for multicellular survival.

Second, if cellular decision making is inherently stochastic and highly context dependent, we need systematic studies to identify how stochasticity itself changes as a function of context. For example, human pluripotent stem cells and undifferentiated cancer cells exhibit much higher stochasticity compared with differentiated unipotent cells (101). As a result, modeling the quantitative input–output relationships of PI3K signaling in pluripotent stem cells is likely to be much more challenging, potentially resulting in lower predictive power. Nevertheless, modern computing and advances in numerical workflows can be harnessed to improve on this, as has been done in the context of an entirely different but conceptually similar field: meteorology (109, 110). Like cells, weather systems also have state-dependent limits on predictability and are highly sensitive to variable initial conditions (*i.e.*, context). Conversely, the constant improvements in weather prediction accuracy rests on the ability of the underlying mathematical models to capture context-dependent changes in noisy processes as a function of time (109). There is therefore a real opportunity for multidisciplinary collaborations to develop quantitative, context-dependent, and noise-aware models of PI3K signaling, building on knowledge transfer from other scientific fields.

The aforementioned considerations prompt entirely novel questions in relation to our understanding of disease-causing PI3K signaling perturbations, including the highly prevalent *PIK3CA* mutations in human cancers and rare overgrowth syndromes. To date, studies of such mutations have mainly focused on average shifts in signaling responses, for example, the well-known increases in average PI3K signaling output in the presence of strongly activating *PIK3CA* mutations. It is now equally pertinent to examine the extent to which disease-associated mutations in PI3K signaling components act by changing the *noise* in a response and how such changes affect the likelihood and persistence of a disease-causing phenotypic cell state.

## Therapeutic implications

Although the last decade has seen the regulatory approval of several targeted inhibitors against components of the PI3K signaling network (111, 112), the road to clinical success has been challenging. The use of the PI3Kα-specific inhibitor alpelisib (BYL719) for treatment of overgrowth disorders or estrogen receptor–positive breast cancers with activating *PIK3CA* mutations is a case in point. Efficacy is limited by cell-intrinsic feedback loops as well as on-target toxicities, in particular disruption of organismal glucose homeostasis. Upon whole-body PI3Kα inhibition, the ensuing hyperglycemia triggers a compensatory increase in pancreatic insulin secretion, which like the cell-intrinsic feedback loops (93, 113, 114, 115, 116), seeks to overcome the pharmacological suppression of PI3K signaling (117). More generally, drug-induced upregulation and downregulation of feedback loops leads to qualitatively distinct outcomes and often unanticipated drug resistance. For example, a computational simulation of a time course of active PI3K, ERK, and S6K upon increasing AKT inhibition predicts different degrees of feedback-mediated RTK activation and thus drug resistance as a function of total FOXO protein levels (118).

A biochemical systems view of PI3K signaling may therefore be of great utility for systems pharmacology where the aim is to use prior knowledge and computational modeling to identify intervention strategies that tune a disease network toward a signaling configuration that once again enables normal function (119, 120). Knowledge of signal encoding may enable specific aspects of the underlying signaling dynamics, and not an essential signal transducer (*e.g.*, PI3Kα), to be used as pharmacological target (121). The goal will be to enable disease-specific targeting, without adverse effects on normal cells and tissues. This requires a minimum of three key objectives to be met.

1. Systematic mapping of the quantitative input–output relationships within the PI3K signaling network across relevant contexts, such as lineage diversity, microenvironmental conditions, and genetic background;
2. A mathematical framework that captures observed signaling states and their temporal evolution according to a probabilistic landscape of cellular decision making (96) (see also the subsection on “The importance of noise in cellular decision making”);
3. Computational models trained on (1) and (2), with the ability to predict therapeutically relevant perturbations capable of stabilizing a desired network configuration and phenotypic function, as determined by the disease context.

Recently, differentiable programs have been proposed as promising solutions to the challenge of modeling phenomena ranging from the small and specific (*e.g.*, an experimental assay) to the broad and complex (*e.g.*, cell fate decisions or protein folding) (122). Such programs represent machine-learnable models that harness knowledge about basic phenomena (*e.g.*, physicochemical constraints and network motifs) to address the challenge of using diverse and typically sparse and noisy experimental data (122). Differentiable programs may therefore prove particularly useful in efforts to develop clinically relevant prediction tools for effective targeting of PI3K-associated disorders.

## Methodological considerations

**Figure 6:**
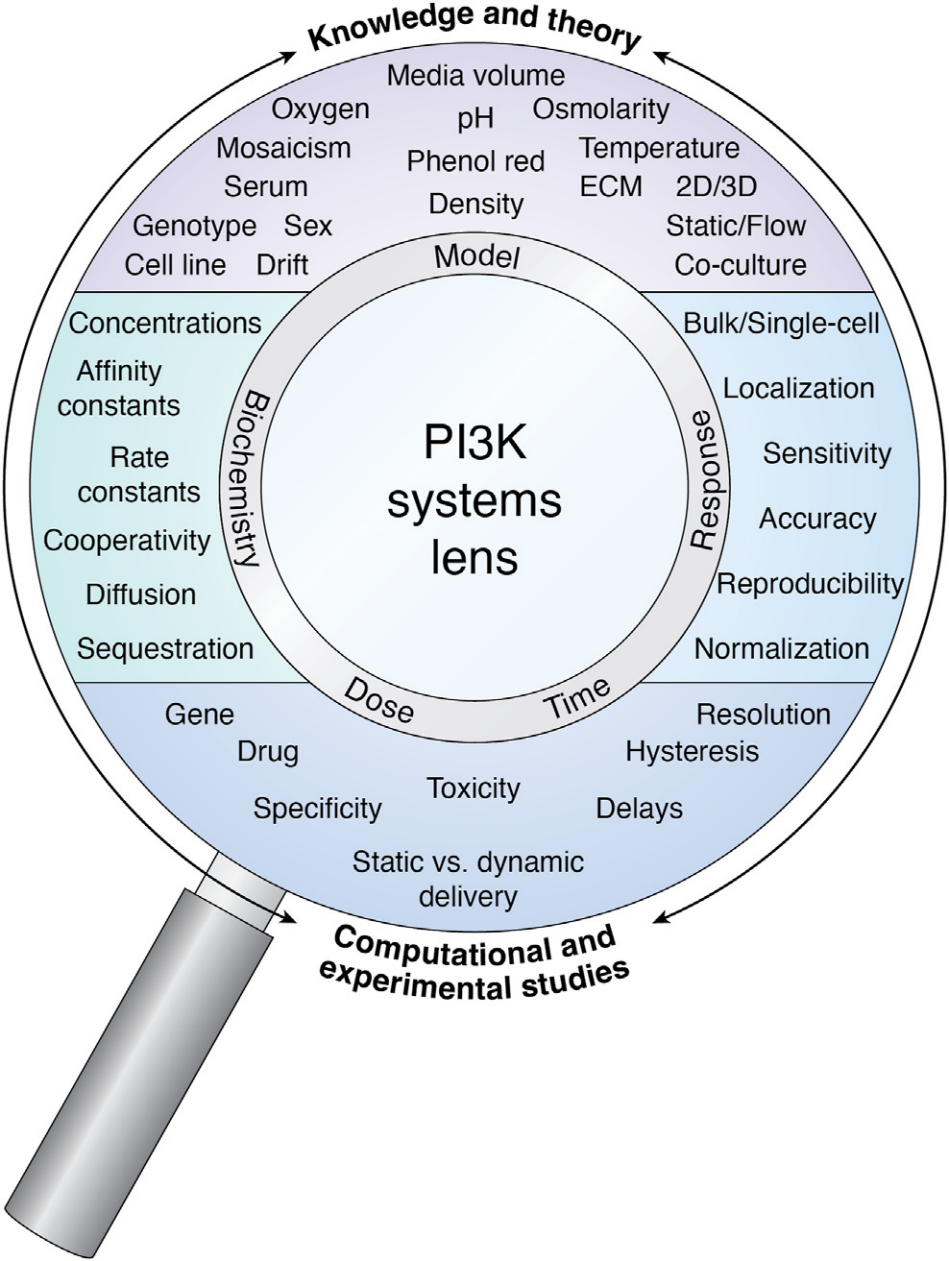
**PI3K signaling through a biochemical systems lens.** A systems biology of PI3K signaling requires a cyclic integration of theoretical knowledge, computational models, and experimental studies. Accurate models of PI3K signaling responses must feature high-quality biochemical measurements, attention to model- and experiment-specific variables, including dose and time. Careful spatiotemporal measurements are needed to resolve the nonlinear complexity of PI3K signaling responses, and how this “code” contributes to reliable information transmission within individual cells and, ultimately, across entire cell populations. Addressing this gap in quantitative PI3K signaling in a time- and cost-efficient manner requires systematic interdisciplinary effort to enable standardized experimentation and downstream data integration.

Achieving a systems biology understanding of the PI3K signaling network calls for an in-depth understanding of and practical proficiency in biophysics, biochemistry, cell biology, physiology, bioinformatics, statistical mechanics, control theory, mathematical modeling, and last, but not least, high-quality data management. Success will therefore depend on interdisciplinary and multidisciplinary collaborations, similar to the approach undertaken by the National Institutes of Health–based LINCS (The Library of Integrated Network-Based Cellular Signatures) (123). This features a common set of agreed workflows that ensure high quantitative precision and technical and analytical reproducibility within and across individual laboratories. In the following, we provide an overview of what we consider to be critical factors that warrant early consideration in any workflow aimed at deciphering the complexity of cellular signaling, with a particular focus on the PI3K network (Fig. 6).

### Biological model system

Experimentalists should invest sufficient time and effort upfront into understanding the pros and cons of choosing particular biological systems for studies of any signaling network. An initial molecular profiling of a cell line at baseline can help parametrize context-dependent signaling models (124, 125) and thus prioritize the set of experimental perturbations that will be most informative for testing or updating model performance. A large number of (mostly cancer) cell lines already have their genomic, transcriptomic, and, to a more limited extent, proteomic profiles publicly available. That said, one should bear in mind the partial dependence of cell line molecular configurations on numerous physicochemical parameters, including cell density (126), general culture set-up (extracellular matrix, 2D *versus* 3D, coculture, adherent *versus* suspension) (127, 128, 129, 130, 131, 132, 133), media composition (including serum origins) (134), and oxygen tension (135), to name a few. It is essential that such factors are considered at the outset and carefully monitored and recorded throughout the course of a study (136, 137). While perfect control may be impossible, paying attention even to subtle variations can greatly facilitate experimental troubleshooting and final data integration, especially when multiple groups contribute to a common project (136). Such attention to detail is even more crucial when studying live-cell signaling responses over long periods given the many potentially confounding factors (138), including growth factor depletion, biological fluorescence, cell movement, and physical perturbations.

Beyond environmental variables, genetic drift may cause even seemingly identical cell lines to behave differently under otherwise similar conditions (139). Although rarely mentioned, it is also essential to consider the sex of the cells and how this may influence the choice of additional culture components such as phenol red and serum (140). All these considerations apply to simple cell models and to more complex organoid and animal systems alike (141). When nonprimate models are used, species-specific differences must also be considered if the goal is to draw inferences of relevance to human physiology and disease (142, 143). Finally, systematic confounders can be minimized with randomization, especially when experiments involve multiwell treatments and measurements (144).

### Gene and drug doses

Invariably, signaling studies seeking to establish cause-and-effect relationships rely on perturbation experiments. Most of these feature genetic and/or pharmacological approaches. To some extent, the choice of these is inherent to the choice of biological model(s), and yet key considerations are worth highlighting separately when it comes to quantitative signaling studies.

First, it is important to avoid the trap of binary thinking, which often leads to the use of genetic overexpression models even if alternatives are available. In the section “Biochemical determinants,” we alluded to the fact that the nonlinear input–output relationships that govern signal transduction networks make these extremely sensitive to subtle changes in specific biochemical determinants, including molecular concentrations. The system-wide consequences of such changes are difficult if not impossible to predict with unaided intuition alone. Given advances in CRISPR–Cas9 and precision gene editing, it is prudent to minimize the use of overexpression despite the faster time to (potentially irrelevant) results afforded by such systems. This is especially important if the aim is to model the consequences of disease-causing variants in PI3K signaling components (145). If overexpression is unavoidable, great care must be taken to ensure that all relevant controls have been considered (146), and where possible, a putative regulatory mechanism should be validated through orthogonal approaches (147). Conversely, the ablation or partial reduction of a molecular target are not free of confounders either, and such approaches must also be accompanied by attention to system-wide effects beyond the intended change. While rescue experiments are conceptually appealing, the existence of hysteresis in a system, defined as the ability to reach different steady states depending on whether a parameter is increasing or decreasing (148), may cause even the most controlled rescue experiment to fail.

The use of drugs as experimental perturbations should also abide by the principles of quantitative biochemistry. The extent to which a drug hits both its target(s) and off-target(s) depends on the parameters of the system under study, including the concentrations of individual target molecules. Consequently, EC_50_ and IC_50_ values are context-specific, and, when available, should be used as guiding for the set-up of dose–response assays to determine the optimal concentration for a different system. Moreover, to avoid misleading conclusions of a binary nature (*e.g.*, the drug has/does not have an effect), both acute and long-term signaling changes should be evaluated in the relevant model system. For example, dose- and time-dependent proteomics can help decrypt drug actions while also identifying nonlinear signal propagation suggestive of crosstalk and/or feedback regulation (149). Finally, given the increasing choice of small-molecule inhibitors with high specificity for PI3K signaling components (111), studies should avoid the use of nonspecific and now largely obsolete first-generation agents such as LY294002 and wortmannin (150, 151, 152, 153, 154).

### Kinetics

Despite, or perhaps as a result of, technical limitations, high-resolution kinetic measurements were the norm in foundational quantitative biochemistry studies seeking to map the functional versatility and potential for signal amplification in multicyclic phosphorylation cascades (155). With the molecular biology revolution, its delivery of The Human Genome Project, and the subsequent rise in -omics technologies, much of this quantitative biochemistry has been lost in favor of increasing the number of perturbation conditions (*e.g.*, drugs and unique cellular contexts). To avoid astronomical expenses and unmanageable experimental complexity, most -omics measurements of PI3K and other signaling markers are therefore mostly representative of isolated snapshots. It is neither appropriate to discount such data altogether nor is it our intention. It is, however, important to be fully aware of the limitations of snapshot signaling studies and how response variables may or may not be detected as a function of time if this parameter features in the experimental design. A snapshot -omics measurement of the PI3K signaling network may generate a lot of dots, yet without a time component, connecting the dots (*i.e.*, understanding how and when information *flows* among them) is impossible. This approach may also result in erroneous conclusions. This is arguably best illustrated with the time delay between AKT activation and mTORC1 activation upon acute growth factor stimulation. Initial studies of growth factor–induced AKT phosphorylation dynamics revealed a 5- to 10-min delay between AKT phosphorylation and subsequent mTORC1-dependent S6 phosphorylation in PC12 cells stimulated with EGF (156). If the authors had relied only on a snapshot measurement after 5 min of stimulation, they would have concluded that EGF triggers AKT but not S6 phosphorylation; the opposite would have been inferred if they had taken a single measurement at any point 10 to 60 min after EGF addition (156). This transduction delay also holds for other cellular contexts and growth factors, and is, in fact, integral to the operation of multicyclic signaling cascades (155, 157).

Time delays and cellular context must also be considered when evaluating crosstalk between PI3K signaling and other parallel pathways such as RAS–ERK signaling. Initial experimental approaches led to the assumption that PI3K–AKT activation opposes RAS–ERK activity (158, 159, 160). In retrospect, however, these early data would be considered insufficient for generalizable conclusions given the technical limitations at the time, with mechanistic data featuring overt overexpression, use of nonspecific inhibitors, and snapshot signaling measurements. A more nuanced view is therefore needed considering numerous other examples of what appears to be a positive reciprocal relationship between PI3K and RAS–ERK activation (161, 162, 163, 164, 165, 166, 167). It is worth noting that these findings are not at odds with the common observation of compensatory activation of RAS–ERK signaling upon inhibition of PI3K signaling, a phenomenon that occurs downstream of a FOXO-mediated transcriptional feedback loop that leads to increased expression of RTKs (Fig. 2).

At present, our understanding of quantitative PI3K signaling flow is most advanced in the context of insulin-stimulated adipocytes, thanks to the application of high-sensitivity phosphoproteomics at high temporal resolution (168). This can now be expanded to additional growth factors and cellular contexts for a systematic mapping of the information transfer functions that govern context-dependent PI3K signaling and crosstalk. The choice of time points must be guided by the biological question, including the response variables of interest (acute signaling *versus* transcriptional responses). A mathematical model may prove particularly useful at the initial stage of experimental design by restricting measurements (and thus cost) to time points that are most likely to be informative for a given question. If possible, a combination of acute and long-term measurements will always yield the most information by enabling disentangling of direct effects from indirect effects as well as nonlinear input–output relationships. How this can be achieved alongside the systematic modulation of signaling dose is illustrated in a study of ERK-induced responses in retinal pigment epithelial cells with inducible expression of the BRAF^V600E^ oncogene (169). Using a combination of live-cell imaging of cell cycle and ERK activity reporters, alongside deep transcriptomic profiling of cells following acute and sustained oncogene induction in the presence of increasing ERK inhibition, this study identified a bell-shaped relationship between ERK activity, distinct transcriptional programs, and cellular proliferation (169). The use of microfluidics devices is another powerful method for measuring kinase signaling activities, transcription factors, and/or target genes at scale and with single-cell resolution (170, 171).

### Resolution

Beyond attention to single- *versus* bulk-cell and spatiotemporal resolution of signaling responses (Fig. 4), attention to resolution also applies to the role of individual protein isoforms and the functional diversity they give rise to as part of multimeric signaling complexes. For example, class IA PI3K is an umbrella term for 15 different heterodimeric enzymes (*i.e.*, five possible regulatory subunit isoforms can combine with either one of three possible catalytic subunit isoforms). Not all 15 constellations are equally likely in a given setting, depending instead on expression levels, relative affinities, and subcellular localization. There is also evidence that the nucleocytoplasmic distribution of catalytic and regulatory subunits of class IA PI3K exhibits isoform-, lineage-, and stimulus-specific differences (172, 173, 174). Moreover, cancer-associated helical-domain *PIK3CA* mutations may exert their effects through a noncatalytic mechanism that involves nuclear translocation of the regulatory subunit isoform p85β but not p85α (175). Thus, there is a clear need for a systematic focus on context-dependent spatiotemporal regulation of specific protein isoforms, starting with key effectors (*e.g.*, AKT1 *versus* AKT2 *versus* AKT3) within the canonical PI3K signaling cascade. To avoid the fragmented nature of the current evidence base, however, we suggest a community effort to develop guidelines and standards for subcellular profiling of PI3K signaling, with a strong emphasis on key controls and orthogonal validations that limit the confounding effects of assay-specific artifacts. An example of the latter may include aggregation artifacts and false-positive subcellular enrichments for certain phosphoinositides if researchers are not familiar with the optimal immunofluorescence protocols for phosphoinositide detection.

Resolution is also relevant when considering signaling crosstalk, in particular between the PI3K–AKT and RAS–ERK networks given their overlapping upstream activators, downstream effectors, and target phenotypes. Joint experimental measurements of signaling markers for both networks could be critical for predictive modeling of cell fate decisions if the latter depend on nonredundant and highly coordinated activation of both signaling branches (106, 176). A quantitatively tractable approach currently in use for such joint profiling in live cells involves the coexpression of genetically encoded biosensors for AKT and ERK activity, with the potential for simultaneous monitoring of individual cell cycle phases (177, 178, 179).

### Response variables

For accurate and quantitative modeling, much upfront effort is needed to obtain a detailed understanding of key response variables, including biochemical properties and technical details. Such details include dynamic range and signal-to-noise ratio, including how these parameters change as a function of the chosen measurement technique. For example, to use immunoblot measurements for accurate quantitative inference, a dilution series of the lysate to generate a mini standard curve for each marker is best practice (180), a method that is already in place for high-throughput reverse-phase protein array analyses (181). Such standardization will also aid with the data normalization that is typically required for direct comparisons between experimental outcomes and model simulations in systems biology signaling studies.

An overview of the available literature may also give the wrong impression that measuring the levels of AKT phosphorylation on thr308 and/or ser473 (AKT1 numbering) is the most reliable method for evaluating proximal PI3K signaling activation. It is therefore worth emphasizing what these and other measurements actually represent, given their wide usage and relevance for accurate signaling inference. In general, a phosphorylation measurement typically reflects the relative balance between the activity of a kinase and that of one or more corresponding phosphatase(s). A change in AKT phosphorylation on thr308 could therefore reflect a change in the activity of 3-phosphoinositide-dependent kinase 1, a change in the activity of the phosphatase PP2A, or both (4, 182). For AKT ser473 phosphorylation, mTORC2 is often considered to be the primary kinase although the exact mechanisms, including the potential for AKT autophosphorylation, may differ depending on cell type and context (183, 184, 185, 186). AKT ser473 dephosphorylation is mediated by PH domain leucine-rich repeat protein phosphatases (4, 187). What should be clear, however, is that AKT phosphorylation is not a measure of AKT activity, a misconception that persists in the literature. In fact, thr308 and ser473 typically become hyperphosphorylated when AKT kinase activity is suppressed with catalytic AKT inhibitors (188, 189, 190). Thus, as a minimum, claims of AKT activation must include measurements of AKT substrate phosphorylation (*e.g.*, PRAS40 pthr246). Similarly, while there is a strong correlation between PI3K activation and AKT phosphorylation, it is far from deterministic, and caution is warranted particularly if one is tempted to conclude that lack of AKT phosphorylation equates to lack of PI3K activity. These considerations apply to most if not all readouts within the PI3K and other signaling networks, including exogenous reporters such as commonly used genetically encoded biosensors (191, 192).

## Conclusion

Readers may wonder whether we, too, are not simplifying complexity too much by referring mostly to PI3K signaling in relation to individual cells, and thus seemingly disregarding the additional complexity that emerges from interactions amongst cellular collectives, particularly in the context of tissues, organs, and even whole organisms. Indeed, as already discussed, the “system” in systems biology was originally reserved for physiology. The ultimate goal is therefore to understand how networks of various types and sizes, both within and across cells, tissues, and organs, acquire the capability of performing complex functions that are inaccessible to and beyond the sum of their parts (193, 194). Yet, as we have highlighted the knowledge about individual parts, when integrated into predictive quantitative models, enables reconstruction and further experimental validation of systems behavior. Thus, while multiscale PI3K systems biology will be highly relevant, for example, for understanding the complex physiology of insulin, one must first account for the operation of PI3K signaling at smaller scales. This is akin to the initial need to identify the individual components of the PI3K signaling network before starting to understand how their interactions in space and time enable individual cells to sense and decode growth factor–specific information.

Complexity in signaling, and biology more generally, therefore appears to be a self-perpetuating continuum, operating across scales. Much like viewing the image of a fractal, whether we zoom in or whether we zoom out, we see a self-similar representation of the original, as determined by a set of common rules that govern the interactions of individual units. It is our conviction that the signaling field, including all relevant technologies, is now sufficiently mature to begin the systematic mapping of the context-dependent cellular rules that govern PI3K-dependent information transmission. It will require those working in the field to make the relevant computational and theoretical concepts part of everyday PI3K signaling studies. How a mindset change of this kind can make the impossible possible is perhaps best illustrated by transformational tools like AlphaFold2 and RoseTTAFold in protein structure prediction (122, 195). We are of the opinion that a similar evolution in our experimental approach will enable transformational leaps in our understanding of PI3K signaling in health and disease. It is also our hope that this piece, alongside several others that helped fuel our own interest in this topic (95, 122, 196, 197, 198, 199, 200), will inspire those working in this field to take the leap.

## Conflict of interest

A. T. is the Editor-in-Chief of the *Journal of Biological Chemistry*. The authors declare that they have no additional conflicts of interest with the contents of this article.
